# Prognostic significance of mucin expression profiles in breast carcinoma with signet ring cells: a clinicopathological study

**DOI:** 10.1186/s13000-016-0584-1

**Published:** 2016-11-15

**Authors:** Ryuji Ohashi, Ayako Hayama, Keiko Yanagihara, Koji Yamashita, Takashi Sakatani, Hiroyuki Takei, Zenya Naito

**Affiliations:** 1 Department of Diagnostic Pathology, Nippon Medical School Hospital, 1-1-5, Sendagi, Bunkyo-ku, Tokyo 113-8603 Japan; 2 Department of Breast Surgery, Nippon Medical School Hospital, 1-1-5, Sendagi, Bunkyo-ku, Tokyo 113-8603 Japan; 3 Department of Integrated Diagnostic Pathology, Nippon Medical School, 1-1-5, Sendagi, Bunkyo-ku, Tokyo 113-8603 Japan

**Keywords:** Signet ring cells, Breast cancer, Mucin

## Abstract

**Background:**

Signet ring cells (SRCs) often accompany gastrointestinal carcinoma, referred to as SRC carcinoma; however, breast cancers containing SRCs have not been well characterized, leaving the prognostic significance of SRCs undetermined. We have described clinicopathological characteristics of patients with breast cancer containing SRCs in relation to the expression levels of MUC1, MUC2, MUC4, MUC5AC, and MUC6.

**Methods:**

Twenty-two breast cancer cases with variable degrees of SRC population were retrospectively studied. Each case was categorized as high (>31 %) or low (<30 %) SRC tumor. The SRCs were morphologically classified into the intra-cytoplasmic lumen (ICL) type, or the non-ICL type. The expression levels of MUC1, MUC2, MUC4, MUC5AC and MUC6 were determined immunohistochemically. Depending on its subcellular localization, MUC1 was categorized as the luminal and cytoplasmic (LC) type, or the cytoplasmic with circumferential membranous accentuation (CM) type. These histological findings were compared with other clinicopathological parameters.

**Results:**

The series consisted of invasive ductal carcinoma (*n* = 9), invasive lobular carcinoma (*n* = 9), and mucinous carcinoma (*n* = 4) cases. The SRC population accounted for 8–81 % of the tumor cells. Eight cases had ICL type SRCs, and the remaining 14 had non-ICL type SRCs. Neither the high (*n* = 12) and low (*n* = 10) percentage of SRCs, nor the SRC types affected the clinicopathological parameters. In the low MUC1 group (*n* = 11), larger tumors, higher nuclear grade, lymph node metastasis, and negativity for estrogen receptor was more frequently identified compared to the high MUC1 group (*n* = 11; *p* = 0.01, *p* = 0.002, *p* = 0.008, and *p* = 0.02, respectively). The CM group (*n* = 7) had more patients with large-sized tumors, lymph node metastasis, lymphovascular invasion, and higher Ki67 indices than the LC group (*n* = 15; *p* = 0.04, *p* = 0.001, *p* = 0.006, and *p* = 0.03, respectively). The expression levels of MUC2, MUC4, MUC5AC, and MUC6 showed no clinicopathological significance. Two patients with low MUC1 expression and CM patterns had tumor recurrence, resulting in death, while all the other patients survived without recurrence.

**Conclusion:**

Our results demonstrate that in breast cancers containing SRCs, low MUC1 expression and/or its CM subcellular localization patterns are associated with unfavorable clinicopathological factors. The utility of MUC1 expression as a prognostic marker remains to be verified in future studies.

## Background

Signet ring cells (SRCs) are types of epithelial cells morphologically characterized by intracytoplasmic mucins displacing the nucleus to one side of the cell. SRCs are often involved in the gastrointestinal carcinoma, referred to as the SRC carcinoma when SRCs constitute a major component of the tumor [1–4]. Saphir first described the presence of SRCs in the breast cancer [5]. However, breast carcinomas predominantly composed of SRCs are rarely encountered in daily clinical practice [6–8]. SRCs of the breast tumors were initially thought to originate from invasive lobular carcinoma (ILC), whereas later studies found that the SRCs can derive from other types of carcinoma besides ILC, including invasive ductal carcinoma of no special type (IDC) and mucinous carcinoma (MC) [9–13]. Likewise, the diagnostic criteria for SRC carcinoma of the breast have not been strictly defined yet, and thus SRC carcinoma has not been listed as a distinct disease entity in the current WHO classification of breast tumors [14]. Therefore, it is imperative to conduct studies that extensively reveal the characteristics and clinical significance of SRCs in breast carcinomas.

Mucins are high molecular weight heavily O-glycosylated glycoproteins produced by various epithelial cells and malignant tumors, labelled with a number reflecting the order in which each mucin was initially discovered [15]. Membrane-bound forms of mucin include MUC1, MUC3A, MUC3B, MUC4, MUC12, MUC13 and MUC17, and the secreted forms include MUC2, MUC5AC, MUC5B and MUC6. The aberrant expression of mucins could be associated with cancer growth, differentiation, transformation, and invasion [16]. In breast cancer, most tumors express MUC1 and MUC3, and the expression of MUC2, MUC4, MUC5AC, and MUC6 is variable or limited [17]. A previous study reported an inverse association between the degree of MUC1 expression, and tumor grade and a recurrence rate [17]. In addition, increased cytoplasmic staining of MUC1 with circumferential membranous accentuation could be related to the aggressive behavior of tumors as MUC1 is normally localized to apical sites of breast ductal epithelium [17–19]. These findings indicate that MUC1 can be a potential prognostic indicator of patients with breast cancer. However, reports on the mucin expression profiles of breast cancer containing SRCs are still limited [20–22]. In particular, the clinical significance of the mucin expression profiles of SRCs in breast carcinoma remains to be further investigated.

In the present study, we investigated the clinicopathological features of 22 patients with breast carcinoma containing SRCs to variable degrees. We divided the patients into high and low SRC groups, comprising more or less than 30 % of the tumor cells, respectively, to assess the effect of SRC population on other clinicopathological features. We also morphologically categorized SRCs into the intra-cytoplasmic lumen (ICL) and non-ICL types, according to the 2013 WHO classification, to see if SRC types may have clinical significance [14]. We further performed immunohistochemical analysis to assess the expression of MUC1, MUC2, MUC4, MUC5AC, and MUC6 to delineate the prognostic significance of the expression level of each mucin with a particular emphasis on the expression level of MUC1 and its subcellular localization pattern.

## Methods

### Case selection

We searched the archives of the Department of Diagnostic Pathology, Nippon Medical School Hospital (Tokyo, Japan) between January 2006 and August 2015. We identified 22 cases with “SRC” in the diagnostic lines and/or histological description. Patients with tumors in the breast metastasized from primary carcinomas in other organs (the gastrointestinal system or the lung) were excluded. We reviewed all the hematoxylin and eosin-stained histological specimens for each case to confirm the presence of SRCs within the tumor. The cytomorphology of SRCs was categorized as the ICL and non-ICL types as described elsewhere [14]. Briefly, the ICL type is characterized by the presence of ICL with targetoid appearance, whereas the non-ICL type has abundant intracytoplasmic mucin as observed in gastric carcinoma. Nuclear atypia was graded by combining four nuclear features including 1) enlargement, 2) distinct nucleolus, 3) hyperchromasia and 4) pleomorphism, and was expressed as grade 1: none or 1 of the features, grade 2: 2 or 3 of the features, and grade 3: all of the features [23]. The population of SRCs in each case was assessed quantitatively by two pathologists (RO and TS) who independently determined the percentage of SRCs among the total number of tumor cells evaluated in at least 20 high power fields focusing on the SRC-rich areas. The cases were further stratified into SRC-high (>31 %) and SRC-low (<30 %) groups.

Clinical data extracted from medical charts and pathology reports were reviewed. This study was conducted in accordance with the principles embodied in the Declaration of Helsinki (revised in Brazil 2013). Patients’ consents were obtained for the use of clinical samples for research purposes according to the regulations defined by the Ethics Committee of Nippon Medical School Hospital.

### Immunohistochemical stainings

Immunohistochemical staining was performed on formalin-fixed histological sections of the tumors with primary antibodies by using the standard avidin-biotin-peroxidase complex technique. The following antibodies were used: monoclonal mouse anti-human MUC-1 (NCL-MUC-1, dilution 1:100; Leica Biosystems Newcastle Ltd, UK), monoclonal mouse anti-human MUC-2 (NCL-MUC-2, dilution 1:100; Leica Biosystems Newcastle Ltd), monoclonal mouse anti-human MUC-4 (1G8, dilution 1:100; Santa Cruz Biotechnology, USA), monoclonal mouse anti-human MUC-5 AC (NCL-MUC-5 AC, dilution 1:100; Leica Biosystems Newcastle Ltd), monoclonal mouse anti-human MUC-6 (NCL-MUC-6, dilution 1:100; Leica Biosystems Newcastle Ltd), E-cadherin (NCH-38, dilution 1:50; DAKO, Denmark), monoclonal rabbit anti-human estrogen receptor (ER) (SP1, dilution 1:1; Ventana Medical Systems, USA), monoclonal rabbit anti-human progesterone receptor (PgR) (1E2, dilution 1:1; Ventana Medical Systems), monoclonal rabbit anti-human HER2/neu (4B5, dilution 1:1; Ventana Medical Systems), and monoclonal mouse anti-human Ki67 (M7240, dilution 1:100; DAKO, Denmark). Positive and negative controls for each antibody were used.

MUC1 positivity was determined using the modified H-score [17, 24]. The score consists of the sum of the percent of tumor cell staining multiplied by an ordinal value corresponding to the intensity level (0 = none, 1 = weak, 2 = moderate, and 3 = strong). With 4 intensity levels, the resulting score range was from 0 (no staining in any of the tumor cells) to 300 (diffuse intense staining in all the tumor cells). The cases were then stratified into two groups below and above the median score as low and high groups, respectively. As previous reports indicated that the altered subcellular localizations of MUC1 is of prognostic significance, the MUC1 localization pattern was further categorized as luminal and/or cytoplasmic without membranous accentuation (LC) pattern or as cytoplasmic with circumferential membranous accentuation (CM) pattern [17–19, 25]. In the assessment of MUC2, MUC5AC, and MUC6 expression, positivity was defined by the detection of positive expression in > 10 % of the tumor cells.

The positivity of ER and PgR expression was graded according to the guidelines established at the St Gallen Consensus Conference [26]. Briefly, ER and PgR status should be judged as positive if any positive cells are detected within the tumor. The HER2 status was assessed according to the guidelines defined by American Society of Clinical Oncology / College of American Pathologists [27]. All immunohistochemical stains were blindly assessed by two pathologists (RO and TS), independently.

### Statistical analysis

All data are shown as mean values ± standard error of the mean (SEM). The chi-square and Fisher’s exact tests were used to analyze associations among clinicopathological variables. Multivariate analysis of prognostic parameters was performed using logistic regression. Statistical analyses were performed using JMP statistical software, version 11 (SAS Institute Inc., Cary, NC, USA). A p-value of < 0.05 was considered statistically significant.

## Results

### Clinicopathological profiles of breast carcinoma in association with SRCs

**Fig. 1:**
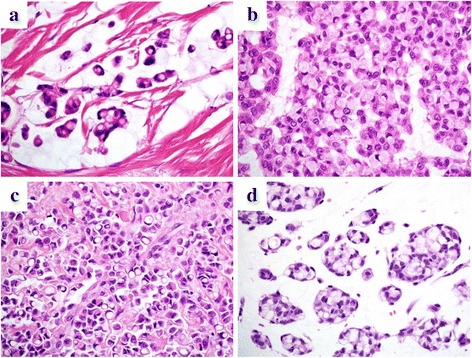
Signet ring cells (SRCs) in invasive ductal carcinoma of no special type (**a** and **b**), invasive lobular carcinoma (**c**), and mucinous carcinoma (**d**). The intracytoplasmic lumen (ICL) type of SRCs is represented by discrete vacuoles with targetoid appearance (**a** and **c**), whereas the non-ICL type has abundant intracytoplasmic mucin dislodging the nucleus to one end of the cells, as seen in gastric carcinoma (**b** and **d**). Hematoxylin and eosin staining (**a**–**d**). Original magnification × 600 (**a**–**d**)

The clinicopathological features of patients are summarized in Table 1. Representative histopathological findings are shown in Fig. 1. The histological variants of the tumors consisted of IDC (*n* = 9), ILC (*n* = 9), and MC (*n* = 4). ILC diagnosis was confirmed by identifying the loss of E-cadherin expression. Patients’ age ranged from 42 to 85 (mean 64 ± 12) years. The tumor size ranged from 0.7 to 6.8 (mean 2.4 ± 1.4) cm. Four cases of IDC, 3 of ILC, and 1 of MC involved grade 2 or 3 nuclear atypia, whereas 5 cases of IDC, 6 of ILC, and 3 of MC had grade 1 nuclear atypia. The SRC population constituted 8 to 81 % (mean 42.2 ± 23) of tumor cells in each case. Tumors of 8 cases had ICL type SRCs, whereas the remaining 14 cases had non-ICL type SRCs. Axillary lymph nodes were positive for metastatic carcinoma in 5 patients with IDC, 3 patients with ILC, and none of the patients with MC. Lymphovascular invasion was observed in 4 patients with IDC, 3 patients with ILC patients, and in none of the patients with MC. None of the patients had distant metastatic carcinoma in other organs at the time of first presentation. With regards to the hormonal status of the tumor cells, 2 cases of IDC, 3 of ILC and 1 of MC showed a triple negative phenotype, 1 patient with IDC showed HER2 type, and all the others showed type A (Table 2). During the clinical follow-up period that ranged from 12 to 137 (mean 49 ± 38) months, two patients (case 3 and case 12) developed breast tumor recurrence in the liver and brain, respectively, and they both eventually passed away. All the other patients survived with no recurrence of the tumor within the follow-up period.

**Table 1:** Clinicopathological variables of patients with breast cancer containing signet ring cells

Case	Age	Tumor site	Histological type	Tumor size (cm)	Nuclear grade	SRC population (%)	SRC type	LN status	LVI	Recurrence	Follow-up (months)	Status
1	57	R-UOQ	IDC	2.1	3	18	Non-ICL	+	+	−	137	Alive
2	42	L-UOQ	IDC	3	1	30	Non-ICL	+	+	−	108	Alive
3	45	L-LIQ	IDC	5	3	80	Non-ICL	+	+	+ liver	66	Dead
4	72	R-UOQ	IDC	2.8	2	21	Non-ICL	−	−	−	67	Alive
5	74	R-UOQ	IDC	2.2	1	39	ICL	−	−	−	30	Alive
6	64	R-UOQ	IDC	2.7	1	30	Non-ICL	+	+	−	12	Alive
7	65	R-UOQ	IDC	0.7	1	55	ICL	+	−	−	45	Alive
8	48	R-LIQ	IDC	2.1	2	79	Non-ICL	−	−	−	12	Alive
9	78	L-LOQ	IDC	1.2	1	81	Non-ICL	−	−	−	12	Alive
10	85	L-IQT	ILC	3.5	1	35	ICL	−	−	−	50	Alive
11	62	R-OQT	ILC	6.8	2	73	ICL	−	−	−	64	Alive
12	80	R-UOQ	ILC	3.1	2	22	ICL	+	+	+ brain	31	Dead
13	57	R-UOQ	ILC	1.8	1	8	Non-ICL	−	−	−	74	Alive
14	47	L-UOQ	ILC	2.8	1	62	Non-ICL	+	+	−	72	Alive
15	55	R-OQT	ILC	2	1	19	ICL	−	+	−	20	Alive
16	76	R-UOQ	ILC	1.2	3	20	Non-ICL	+	−	−	14	Alive
17	52	R-LOQ	ILC	1	1	28	ICL	−	−	−	12	Alive
18	76	L-UQT	ILC	1.8	1	47	ICL	−	−	−	12	Alive
19	78	R-OQT	MC	1.5	1	14	Non-ICL	−	−	−	125	Alive
20	63	R-LOQ	MC	0.7	1	65	Non-ICL	−	−	−	79	Alive
21	66	L-UOQ	MC	1.5	1	62	Non-ICL	−	−	−	35	Alive
22	68	R-OQT	MC	2.5	2	42	Non-ICL	−	−	−	16	Alive

*Abbreviations*: *SRC* signet ring cell, *LN* lymph node, *LVI* lymphovascular invasion, *R* right, *L* left, *UOQ* upper outer quadrant, *LOQ* lower outer quadrant, *LIQ* lower inner quadrant, *IQT* inner quadrant transition, *OQT* outer quadrant transition, *UQT* upper quadrant transition, *IDC* invasive ductal carcinoma, *ILC* invasive lobular carcinoma, *MC* mucinous carcinoma, *ICL* intracytoplasmic lumen

**Table 2:** Immunohistochemical profile of breast cancers containing signet ring cells

Case	MUC1		MUC2	MUC4	MUC5	MUC6	ER	PgR	HER2	Ki67
	Subcellular localization	Level								
1	CM	Low	−	−	−	−	+	+	−	30
2	LC	Low	−	−	−	+	+	+	−	10
3	CM	Low	−	+	+	+	−	−	−	20
4	LC	Low	−	−	+	−	−	−	+	10
5	LC	High	−	−	−	−	+	−	−	5
6	CM	Low	−	−	−	−	+	−	−	30
7	CM	Low	−	−	−	−	+	+	−	30
8	LC	Low	−	+	−	−	−	−	−	5
9	LC	High	−	−	−	+	+	+	−	10
10	LC	High	−	−	−	+	+	+	−	10
11	CM	Low	−	−	−	−	−	−	−	10
12	CM	Low	−	−	−	+	−	−	−	10
13	LC	High	−	−	−	+	+	+	−	10
14	CM	High	−	−	−	−	+	+	−	10
15	LC	High	−	−	−	+	+	+	−	20
16	LC	Low	−	−	−	−	−	−	−	20
17	LC	High	−	−	−	+	+	+	−	5
18	LC	High	−	−	−	−	+	+	−	5
19	LC	High	−	−	−	−	+	+	−	10
20	LC	High	+	−	−	+	−	−	−	10
21	LC	High	−	−	−	−	+	+	−	5
22	LC	Low	+	−	−	+	+	−	−	5

*Abbreviations*: *CM* cytoplasmic with circumferential accentuation pattern, *LC* luminal and cytoplasmic pattern

The association between the SRC population and types and the clinicopathological features are summarized in Table 3. In a total of 12 patients, 5 with IDC, 4 with ILC, and 3 with MC had SRC-high (>31 %) populations whereas 10 patients, 4 with IDC, 5 with ILC, and 1 with MC had SRC-low (<30 %) populations. There was no significant association between the SRC population groups and any of the clinicopathological parameters examined. The non-ICL type of SRC was observed in 7 cases of IDC and all the cases of MC (Fig. 1b, d). By contrast, the ICL type was observed in 6 cases of ILC and 2 cases of IDC (Table 3, *p* < 0.035) (Fig. 1a, c). These findings are consistent with a previous report, which demonstrates that the ICL type SRCs are more commonly identified in ILC than in IDC [28]. There was no significant association between the SRC types and any of the clinicopathological parameters investigated (Table 3).

**Table 3:** Association of SRC population and types with clinicopathological profile

	SRC population			SRC type		
	<30 % (*n* = 10)	>31 % (*n* = 12)		ICL (*n* = 8)	Non-ICL (*n* = 14)	
Age (years)						
> 65	4	7	0.39	5	6	0.37
< 64	6	5		3	8	
Histological type						
IDC	4	5	0.59	2	7	0.035
ILC	5	4		6	3	
MC	1	3		0	4	
Tumor size (cm)						
> 2.1	5	7	0.69	4	8	0.75
< 2.0	5	5		4	6	
Nuclear grade						
1	6	8	0.94	6	8	0.7
2	2	2		1	3	
3	2	2		1	3	
LN status						
Positive	5	3	0.22	2	6	0.4
Negative	5	9		6	8	
LVI						
Positive	5	2	0.09	2	5	0.6
Negative	5	10		6	9	
ER						
Positive	7	8	0.87	6	9	0.6
Negative	3	4		2	5	
TN vs other types						
TN	2	4	0.48	2	4	0.86
Other types	8	8		6	10	
Ki67 (%)						
> 20	4	2	0.22	2	4	0.86
< 19	6	10		6	10	
Recurrence						
Positive	1	1	0.89	1	1	0.67
Negative	9	11		7	13	

*Abbreviations*: *SRC* signet ring cell, *ICL* intracytoplasmic lumen, *IDC* invasive ductal carcinoma, *ILC* invasive lobular carcinoma, *MC* mucinous carcinoma, *LN* lymph node, *LVI* lymphovascular invasion, *TN* triple negative

### MUC1 subcellular localization patterns and expression levels in association with clinicopathological variables

**Fig. 2:**
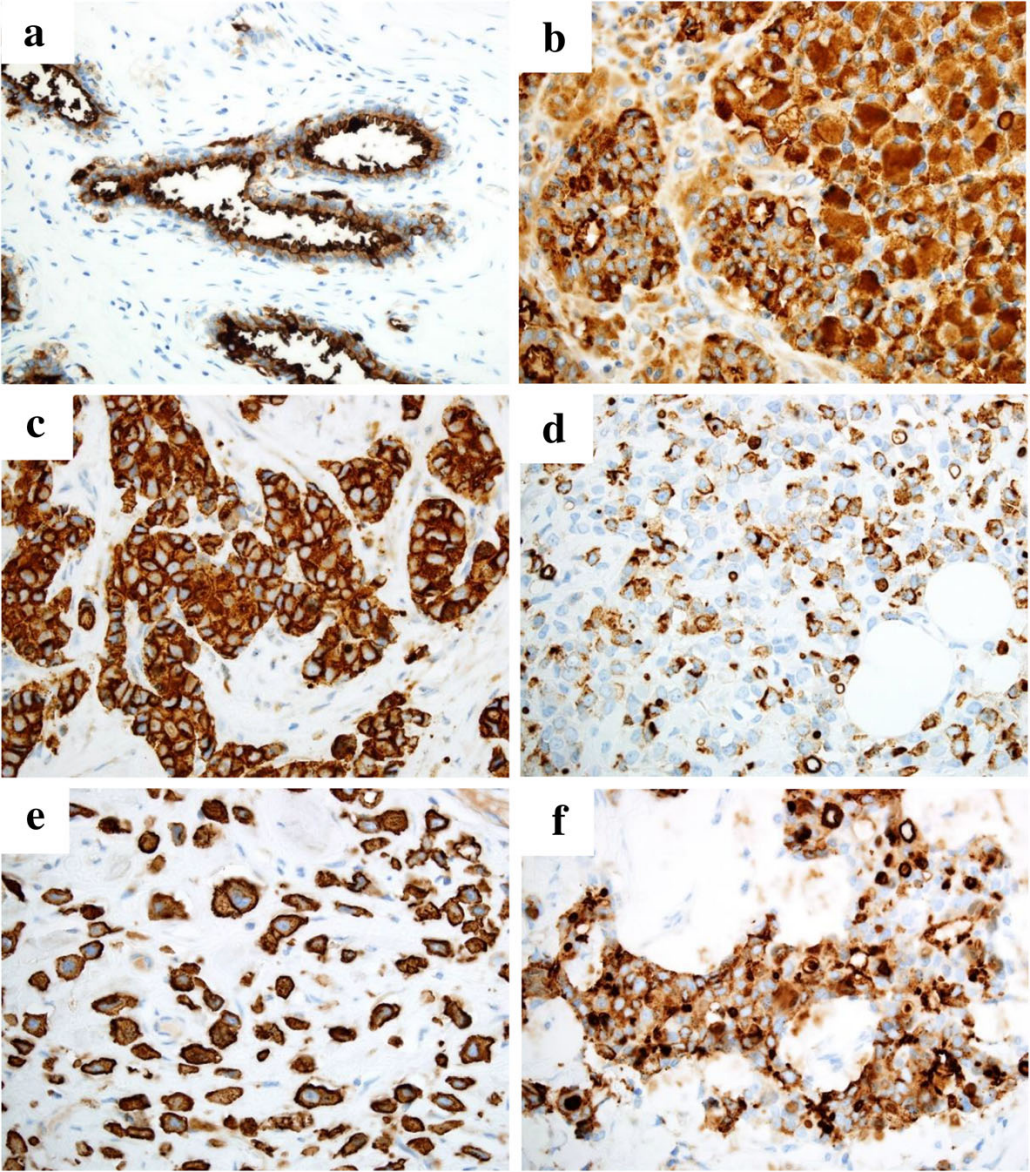
Immunohistochemical expression of MUC1 (**a**–**f**). In normal breast tissue, MUC1 is constitutively expressed in the apical and luminal sites of the ductal epithelia with weak cytoplasmic positivity (**a**). MUC1 was notably expressed in invasive ductal carcinoma (**b** and **c**) and invasive lobular carcinoma (**d** and **e**) either in a luminal and cytoplasmic pattern (**b** and **d**) or in a cytoplasmic with membranous accentuation pattern (**c** and **e**). All mucinous carcinoma cases showed the luminal and cytoplasmic pattern (**f**). Original magnification × 400 (**a**) and × 400 (**b**–**f**)

MUC1 subcellular localization patterns (LC or CM) and expression levels (high or low), and their association with the clinicopathological variables are shown in Tables 2 and 4, respectively. Representative immunohistochemical findings of MUC1 for each localization pattern are presented in Fig. 2. The LC pattern was observed in 15 cases, while the CM pattern was observed in 7 cases. Six patients in the CM group had large tumor sizes, whereas 9 from the LC group had small sizes (*p* = 0.04). Metastatic carcinoma was identified in axillary lymph nodes of 6 patients from the CM group, and 2 patients from the LC group (*p* = 0.001). Five patients of CM group had lymphovascular invasion of the tumor, which was absent in the majority (*n* = 13) of the patients in the LC group (*p* = 0.006). High Ki67 indices (>20 %) were noted in 4 patients in the CM group; however, most (*n* = 13) of the LC group had low Ki67 indices (<19 %) (*p* = 0.03). Two patients in the CM group had tumor recurrences, whereas none of the patients in the LC group did (*p* = 0.03). However, multivariate analysis revealed that MUC localization pattern was not independently responsible for an increase in recurrence rate (*p* = 0.98). There was no significant correlation between the subcellular MUC1 expression patterns and the other variables including age, histological types, SRC population, SRC types, nuclear grade, and hormonal status.

**Table 4:** Association of MUC1 expression levels and subcellular localization patterns with clinicopathological profile

	MUC1 subcellular localization			MUC1 expression level		
	LC (*n* = 15)	CM (*n* = 7)		Low (*n* = 11)	High (*n* = 11)	
Age (years)						
> 61	7	4	0.65	5	6	0.66
< 60	8	3		6	5	
Histological type						
IDC	5	4	0.28	7	2	0.09
ILC	6	3		3	6	
MC	4	0		1	3	
SRC (%)						
> 31	8	4	0.87	5	7	0.39
< 30	7	3		6	4	
SRC type						
Non-ICL	10	4	0.67	8	6	0.38
ICL	5	3		3	5	
Tumor size (cm)						
> 2.1	6	6	0.04	9	3	0.01
< 2.0	9	1		2	8	
Nuclear grade						
1	11	3	0.28	3	11	0.002
2	3	2		5	0	
3	1	2		3	0	
LN status						
Positive	2	6	0.001	7	1	0.008
Negative	13	1		4	10	
LVI						
Positive	2	5	0.006	5	2	0.17
Negative	13	2		6	9	
ER						
Positive	11	4	0.45	5	10	0.02
Negative	4	3		6	1	
TN vs other types						
TN	3	3	0.26	5	1	0.055
Other types	12	4		6	10	
Ki67 (%)						
> 20	2	4	0.03	5	1	0.055
< 19	13	3		6	10	
Recurrence						
Positive	0	2	0.03	2	0	0.14
Negative	15	5		9	11	

*Abbreviations*: *LC* luminal and cytoplasmic pattern, *CM* cytoplasmic with circumferential accentuation pattern, *SRC* signet ring cell, *ICL* intracytoplasmic lumen, *IDC* invasive ductal carcinoma, *ILC* invasive lobular carcinoma, *MC* mucinous carcinoma, *LN* lymph node, *LVI* lymphovascular invasion, *TN* triple negative

High expression of MUC1 was detected in 11 cases, while the remaining 11 cases had low MUC1 expression. Nine patients in the group with low MUC1 expression had larger tumors, whereas 8 patients in the high MUC1 expression group had small-sized tumors (*p* = 0.01). Eight patients in the low MUC1 group had nuclear grades of either 2 or 3; however, all the patients in the high MUC1 expression group had a nuclear grade of 1 (*p* = 0.002). Seven patients in the group with low MUC1 expression had metastatic carcinoma in the axillary lymph nodes, whereas only 1 patient had a positive lymph node in the high MUC1 group (*p* = 0.008). Lymphovascular invasion was identified in 5 patients of the group with low MUC1 expression, while it was observed only in 2 patients of the high MUC1 expression group; however, the difference was not statistically significant (*p* = 0.17). In the group with low MUC1 expression, 6 cases were negative for ER, while 10 cases were positive for ER in the group with high MUC1 expression (*p* = 0.02). Triple negative type cancer was observed in 5 cases of the group with low MUC1 expression, whereas only 1 case in the group with high MUC1 expression exhibited this phenotype with borderline significance (*p* = 0.055). Five patients in the low MUC1 expression group had high Ki67 indices; however, most of the patients (*n* = 10) in the high MUC1 group had low Ki67 indices with borderline significance (*p* = 0.055). Two patients from the low MUC1 expression group had recurrences of the tumor within the follow-up period, whereas none of the patients in the group with high MUC1 expression did, although the difference was not statistically significant (*p* = 0.14). There was no association between MUC1 expression levels and other variables including age, histological types, SRC population, and SRC types.

### The expression of MUC2, MUC4, MUC5AC and MUC6 in association with clinicopathological variables

**Fig. 3:**
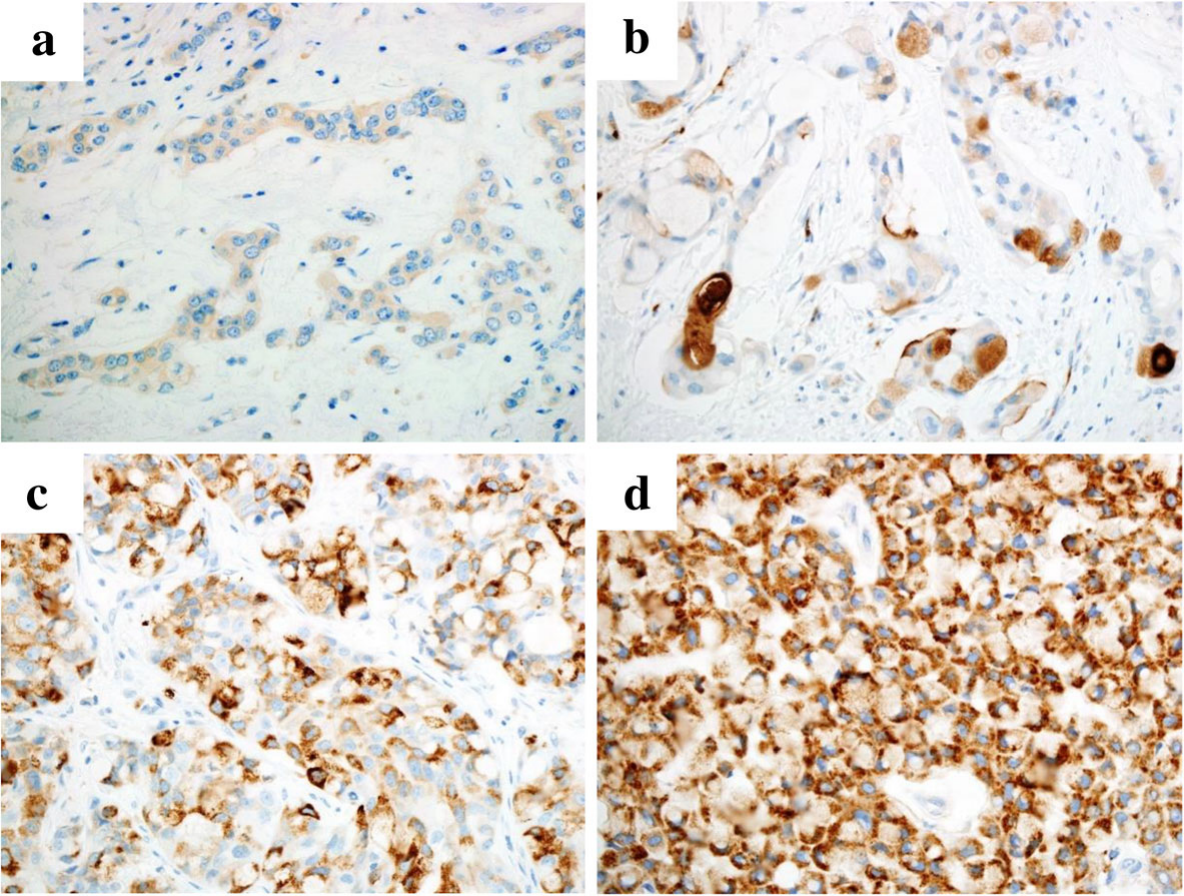
Immunohistochemical expression of MUC2 (**a**), MUC4 (**b**), MUC5AC (**c**) and MUC6 (**d**). Focal weak staining of MUC2 was observed in the cytoplasm of mucinous carcinoma cells (**a**). Positive expression of MUC4 was observed in invasive ductal carcinoma cells including intracytoplasmic mucin (**b**). Conspicuous expression of MUC5AC and MUC6 was noted in invasive ductal carcinoma (**c**) and mucinous carcinoma (**d**), but not in intracytoplasmic mucin. Original magnification × 400 (**a**–**d**)

The expression patterns of MUC2, MUC4, MUC5AC and MUC6, and their representative immunohistochemical findings are shown in Table 2 and Fig. 3 respectively. MUC2 expression was detected in 2 MC cases, but not in other cases. The expression of MUC4 and MUC5AC was detected in 2 IDC cases, one of which showed both MUC4 and MUC5AC expression. MUC6 expression was identified in 10 patients, including 3 with IDC, 5 with ILC, and 2 with MC. The expression levels of MUC2, MUC4, MUC5AC, and MUC6 did not show a significant correlation with any of the clinicopathological variables including SRC population and SRC types.

## Discussion

There have been some controversies concerning the definition of SRCs and the SRC carcinoma of the breast in the last decades. Previous reports indicated that SRCs are originally derived from ILC; however, subsequent studies found that SRCs can originate from other types of breast cancers such as IDC and MC [9–13]. In accordance with these reports, our series of cases consisted of a mixture of ILC, IDC, and MC cases, suggesting that SRC can appear in any kind of breast carcinoma. The 2013 WHO classification classified SRCs as the ICL type with targetoid appearance and the non-ICL type with abundant intracytoplasmic mucin as observed in gastric carcinoma; however, the clinical significance of SRC types remains uncertain [14]. In order to address this issue, we compared cases with ICL and non-ICL types; however, we did not observe significant differences in clinicopathological parameters. Furthermore, the SRC types did not correlate with any of the MUC protein expression tested. These findings indicate that classifying SRC into two morphological types may have little or no clinical significance.

A few earlier studies indicated that tumors with SRC populations constituting more than 10–20 % of the tumor cells are associated with poor prognosis [9, 29]. Nevertheless, the role of SRCs as an adverse prognostic factor for breast cancer has not gained universal acceptance due to the absence of sufficient evidence. In the present study, we stratified the cases as SRC-high (>31 %) and SRC-low (<30 %) groups, depending on the proportion of the SRC population among the tumor cells, and found no significant correlation with any of the clinicopathological variables. In fact, we have tested several thresholds including 40 % or 10 % to divide the cases; however, we were unable to obtain any significant results (data not shown). Based on these results, we suggest that the proportion of SRC population within the breast tumor might not confer a prognostic significance for breast cancer patients. However, these results should be interpreted with caution, considering the fact that our study was conducted using 22 cases with a relatively short follow-up period. Future studies with a large number of cases and a longer follow-up periods are required to further elucidate the clinical significance of SRCs.

MUC1 is normally present on the apical surface of the secretory epithelium; however, its expression levels and cellular localization are altered in malignant tumors [19]. The clinical significance of MUC1 expression in breast cancer has been controversial due to the conflicting results. Rakha et al. and others showed that the presence of MUC1 at the apical cellular site of the ductal epithelium is an indicator of intact MUC-pathway associated with good prognosis, whereas aberrant patterns of expression are indicators of defective MUC1 pathway associated with worse prognosis [17–19, 25]. By contrast, a few prior studies concluded that MUC1 expression levels and/or subcellular localization patterns are not of prognostic significance [30, 31]. In the present study, we showed that lower MUC1 expression levels and altered subcellular localization patterns are associated with adverse clinicopathological variables. However, multivariate analysis did not find a significant association between MUC1 expression pattern and an increase in recurrence rate. As our study involves 22 patients, of whom only 2 had recurrence, the utility of MUC1 expression as a predictor of clinical outcomes warrants a further study using a significant number of cases.

MUC2 is a major secretory glycoprotein produced mainly by the gastro-intestinal epithelial cells [32]. A previous study attributes the less aggressive behavior of MC to the function of MUC2, which is frequently expressed in MC [33]. Our findings are consistent with this study as indicated by positive MUC2 expression in half of the MC cases. The epithelial cells secrete MUC4 as a heterodimer through a proteolytic cleavage mechanism, and the function of MUC4 is to protect vulnerable epithelia. Only a few reports have thus far addressed the significance of MUC4 in breast cancer. A study by Rahka et al. reported MUC4 expression in 95 % of the breast cancer cases in their series, and also detected a correlation with the nuclear grade [17]. In our series, we only observed MUC4 expression in 2 IDC cases, without any association to clinicopathological parameters. MUC5AC and MUC6 share similar functions in protecting the mucosal membrane; however, reports on their expressions in breast cancer are limited. In our study, only one case showed significant expression of MUC5AC, consistent with a previous study that showed MUC5AC expression in < 10 % of breast cancer cases [34]. Another previous study reported MUC6 expression in approximately 23 % of IDC cases examined, and a separate study found association of higher expression of MUC6 with a good prognosis [17, 34]. On the other hand, we detected significant MUC6 expression in 10 cases (45 %) including 3 IDC, 5 ILC and 2 MC cases. The proportion of cases with MUC6 expression in our series is similar to a recent report that shows MUC6 expression in 40 % of breast carcinoma cases; however, an association between MUC6 expression and the clinicopathological variables was not identified [22].

## Conclusion

In summary, our results indicate that instead of SRC population and types, the expression level of MUC1 and/or the presence of cytoplasmic staining with circumferential membranous accentuation pattern in breast cancers showed significant association with adverse clinicopathological parameters. The expression levels of MUC2, MUC4, and MUC5AC had no clinical implication. The utility of MUC1 expression as a prognostic indicator remains to be further assessed using a larger number of cases.

## Abbreviations

CM: Cytoplasmic with circumferential membranous accentuation
ER: Estrogen receptor
ICL: Intracytoplasmic lumen
IDC: Invasive ductal carcinoma of no special type
ILC: Invasive lobular carcinoma
LC: Luminal and/or cytoplasmic
MC: Mucinous carcinoma
PgR: Progesterone receptor
SRCs: Signet ring cells
